# Cost-Effectiveness of a Chemoprophylactic Intervention with Single Dose Rifampicin in Contacts of New Leprosy Patients

**DOI:** 10.1371/journal.pntd.0000874

**Published:** 2010-11-02

**Authors:** Willemijn J. Idema, Istvan M. Majer, David Pahan, Linda Oskam, Suzanne Polinder, Jan Hendrik Richardus

**Affiliations:** 1 Department of Public Health, Erasmus MC, University Medical Center Rotterdam, Rotterdam, The Netherlands; 2 Rural Health Program, The Leprosy Mission Bangladesh (formerly DBLM), Nilphamari, Bangladesh; 3 KIT (Royal Tropical Institute), KIT Biomedical Research, Amsterdam, The Netherlands; University of Oklahoma Health Sciences Center, United States of America

## Abstract

**Background:**

With 249,007 new leprosy patients detected globally in 2008, it remains necessary to develop new and effective interventions to interrupt the transmission of *M. leprae*. We assessed the economic benefits of single dose rifampicin (SDR) for contacts as chemoprophylactic intervention in the control of leprosy.

**Methods:**

We conducted a single centre, double blind, cluster randomised, placebo controlled trial in northwest Bangladesh between 2002 and 2007, including 21,711 close contacts of 1,037 patients with newly diagnosed leprosy. We gave a single dose of rifampicin or placebo to close contacts, with follow-up for four years. The main outcome measure was the development of clinical leprosy. We assessed the cost effectiveness by calculating the incremental cost effectiveness ratio (ICER) between the standard multidrug therapy (MDT) program with the additional chemoprophylaxis intervention versus the standard MDT program only. The ICER was expressed in US dollars per prevented leprosy case.

**Findings:**

Chemoprophylaxis with SDR for preventing leprosy among contacts of leprosy patients is cost-effective at all contact levels and thereby a cost-effective prevention strategy. In total, $6,009 incremental cost was invested and 38 incremental leprosy cases were prevented, resulting in an ICER of $158 per one additional prevented leprosy case. It was the most cost-effective in neighbours of neighbours and social contacts (ICER $214), slightly less cost-effective in next door neighbours (ICER $497) and least cost-effective among household contacts (ICER $856).

**Conclusion:**

Chemoprophylaxis with single dose rifampicin given to contacts of newly diagnosed leprosy patients is a cost-effective intervention strategy. Implementation studies are necessary to establish whether this intervention is acceptable and feasible in other leprosy endemic areas of the world.

## Author Summary

In 2008, 249,007 new leprosy patients were detected in the world. It therefore remains necessary to develop new and effective interventions to interrupt the transmission of *M. leprae*. We assessed the economic benefits of single dose rifampicin (SDR) for contacts as chemoprophylactic intervention in the control of leprosy. The study is based on a large trial including 21,711 contacts of 1,037 patients with newly diagnosed leprosy. We gave a single dose of rifampicin or placebo to contacts and followed them up for four years. The main outcome measure was the development of clinical leprosy. The cost effectiveness was expressed in US dollars per prevented leprosy case. Chemoprophylaxis with SDR for preventing leprosy among contacts of leprosy patients is cost-effective at all contact levels and thereby a cost-effective prevention strategy. In total $6,009 was invested and 38 leprosy cases were prevented after 2 years, costing $158 per prevented leprosy case. Implementation studies are necessary to establish whether this intervention is acceptable and feasible in other leprosy endemic areas of the world.

## Introduction

Leprosy is a chronic infectious disease, caused by the bacillus *Mycobacterium leprae*, which affects the skin and peripheral nerves leading to skin lesions, loss of sensation, and nerve damage. This in turn can lead to secondary impairments or deformities of the eyes, hands and feet. For treatment purposes, leprosy is classified as either paucibacillary (PB) or multibacillary (MB) leprosy. The standard treatment for leprosy is multidrug therapy (MDT) [1]. PB patients are treated for 6 months with dapsone and rifampicin; MB patients are treated for 12 months with dapsone, rifampicin and clofazamine.

The World Health Organisation (WHO) had set a goal in the early 1990s to eliminate leprosy as a public health problem by the year 2000. Elimination was defined as reducing the global prevalence of the disease to less than 1 case per 10 000 population [2]. The WHO elimination strategy was based on increasing the geographical coverage of MDT and patients' accessibility to the treatment. The expectation existed that reduction in prevalence through expanding MDT coverage would eventually also lead to reduction in incidence of the disease and ultimately to elimination in terms of zero incidence of the disease. An important assumption underlying the WHO leprosy elimination strategy was that MDT would reduce transmission of *M. leprae* through a reduction of the number of contagious individuals in the community [3]. Unfortunately, there is no convincing evidence for this hypothesis [4].

With a total of 249 007 new patients detected globally in 2008 [5], it remains necessary to develop new and effective interventions to interrupt the transmission of *M. leprae*. BCG vaccination against tuberculosis offers some but not full protection against leprosy and in the absence of another more specific vaccination against the bacillus other strategies need to be developed, such as preventive treatment (chemoprophylaxis) of possible sub-clinically infected people at risk of developing leprosy. Recently, the results were published of randomised controlled trial into the effectiveness of single dose rifampicin (SDR) in preventing leprosy in contacts of patients [6]. It was shown that this intervention is effective at preventing the development of leprosy at two years and that the initial effect was maintained afterwards.

In order to assess the economic benefits of SDR as an intervention in the control of leprosy, we performed a cost-effectiveness analysis. We provide an overview of the direct costs of this new chemoprophylaxis intervention and calculate the cost-effectiveness compared to standard MDT provision only.

## Methods

### Study population

This study was based on the results of the prospective (sero-) epidemiological study on contact transmission and chemoprophylaxis in leprosy (COLEP; ISRCTN 61223447), which was conducted in the Rangpur and Nilphamari districts of northwest Bangladesh between 2002 and 2007 by the Rural Health Program (RHP) of The Leprosy Mission Bangladesh. The population of the two districts was 4.4 million in 2002, and the number of newly detected leprosy cases in 2002 was 1 317 [7]. Of these, 1 037 patients were included in the COLEP study; 400 with single lesion PB leprosy, 342 with PB leprosy of 2–5 lesions, and 295 with MB leprosy. Intake started in June 2002. Contacts were categorised according to their physical distance to the index patient [8]. For physical distance we defined six categories on the basis of the local housing situation: shares a house only (R) or a house and a kitchen (KR), shares a kitchen only (K), next-door neighbours (N1), neighbours of the neighbours (N2), and social (S) contacts (business contacts and colleagues staying in the same room for at least four hours a day, five days a week).

### Study design

The COLEP study was a single centre, double blind, cluster randomised, placebo controlled trial. A complete description of the COLEP trial is given by Moet *et al.* [6]. In short, at intake - that is, after the index patient had received the second supervised dose of MDT – all contacts of one patient received prophylactic treatment, which included either capsules with 150 mg rifampicin or identical placebo capsules without an active (antibiotic) ingredient. According to bodyweight and age, each contact took two to four capsules under direct supervision of a staff member. Of the 1 037 patients, 517 were allocated to the intervention arm and 520 to the placebo arm of the trial. The number of contacts in the intervention arm was 10 857 and 10 854 in the placebo arm. A follow-up investigation took place two years after intake, starting in June 2004, and completed in February 2006. The primary outcome of the trial was the development of clinical leprosy.

### Ethical clearance

Ethical clearance was obtained from the Ethical Review Committee of the Bangladesh Medical Research Council in Dhaka (ref. no. BMRC/ERC/2001–2004/799). All subjects were informed verbally in their own language (Bangla) about the study and invited to participate. Written consent was requested from each adult. For children consent from a parent or guardian was given.

### Cost calculations

Cost calculations were done from the health care perspective, in which real medical costs were calculated for the chemoprophylaxis intervention compared to the standard MDT treatment program. All direct medical costs of the general health program and the related indirect costs (e.g. transport) for the period 2002–2004 were included. Real medical costs were calculated by multiplying the volumes of health care use with the corresponding unit prices. The calculations of the full cost of the standard MDT treatment program and the rifampicin chemoprophylaxis were based on real resources. If information on resource use and the full cost were available, bottom-up calculations were performed [9]. If detailed information about resource use and unit costs were not available, top-down calculations were performed [10]. Prevention of disabilities, patients' costs and costs caused by loss of production due to absence from work were not taken into account because no reliable data were available. Table 1 provides an overview of the cost calculation method and data source per cost category. All costs were converted into unit prices in US dollars per volume, using the exchange rates of the UN for Bangladeshi taka (BDT) in US dollars ($) [11].

**Table 1:** Calculation method and data source per cost category for the full cost analysis of the standard MDT treatment program.

Cost category	Calculation method	Data source
Program*:		
Personnel	Top-down	Annual Reports DBLM [12], [13]
Transport	Top-down	Annual Reports DBLM [12], [13]
Overhead	Top-down	Annual Reports DBLM [12], [13]
Medical:		
Treatment of leprosy		
MDT**	Top-down	Novartis Foundation [14]
Treatment of complications:		
Reaction type 1	Bottom-up	DBLM- Field guidelines [18]
Reaction type 2	Bottom-up	DBLM- Field guidelines [18]
Surgical intervention***	Top-down	Annual Reports DBLM [12], [13]

The costs (in US$) represent actual expenditures on materials and services obtained.

*Full cost price per newly detected cases among contacts.

**Per month per patient receiving a MDT blister-pack. Based on $ 37 million production cost and $ 3 million on transportation with a production of 32 million blister-packs, for MB 12 blister packs and for PB 6 blister packs are needed, costs $ 15 and $ 7.50, respectively.

***Per patient involved.

The costs of the standard MDT treatment program consisted of the leprosy costs made by the RHP in 2004, including all program costs such as personnel costs (salaries, allowances and staff benefits for all administrative, financial and field staff), transportation costs, surveys (contact and village), overhead costs (administration, repair and maintenance, health education). All data were adjusted for the leprosy share only of the RHP program and based on costs of 2002 and 2003 [12], [13], and extrapolated to 2004 with the corresponding inflation rate [11] because no reliable detailed information was available for 2004. The treatment for leprosy was based on data of the Novartis Foundation for Sustainable Development, which supplies the MDT free of charge [14]. Costs of surgical interventions were based on reconstructive surgery and corresponding hospital costs of 2002. For the treatment of complications all medical intervention and hospital costs were included, based on the annual report of the RHP. The number of patients needing complication treatment was calculated according to the Bangladesh Acute Nerve Damage (BAND) study [15]–[17]. Information from this study was necessary because no data were available for the number of leprosy patients with complications in the COLEP trial. The BAND study was a prospective cohort study of 2 664 new leprosy cases from the same area and the same population as the COLEP study. Over a period of three years incidence rates were calculated with the number of patients developing the following complications: nerve function impairment, type 1 and type 2 reactions, and silent neuritis. Recorded complication rates in the BAND study were extrapolated to the COLEP cohort. The various complications require different treatment regimens, e.g. mild type 1 reaction requires less corticosteroids than severe type 1 reaction. The appropriate treatment for each complication and the associated costs were based on the guidelines for leprosy treatment current at the time of the study [18]. Expert clinical opinion was taken for the average number of mild or severe reaction types. For reversal reactions (RR or Type 1) a distribution of 50% mild and 50% severe reactions was taken, and for erythema nodosum leprosum (ENL or Type 2) per leprosy patient having a reaction an average of two mild and two severe recurrences were taken, costing $ 3.41 and $ 69.58 per patient involved, respectively.

The chemoprophylaxis intervention is additional to the standard MDT treatment program. Therefore, costs for the intervention consist of two components, the basic health care (represented by the standard treatment) and the intervention costs. Therefore, no extra costs for basic health care were added since the intervention is additional to existing practice. For the cost of chemoprophylaxis, COLEP costs were used based on the cost statements of the COLEP Research Study 2002–2004. The chemoprophylaxis costs were calculated bottom-up from the COLEP data base by multiplying the cost for the mean number of capsules ($ 0.21 for 3.5 capsules) with the number of contacts in the intervention. Other medical costs consisted of MDT for all newly detected leprosy patients and treatment of complications.

### Cost-effectiveness analysis

**Figure 1:**
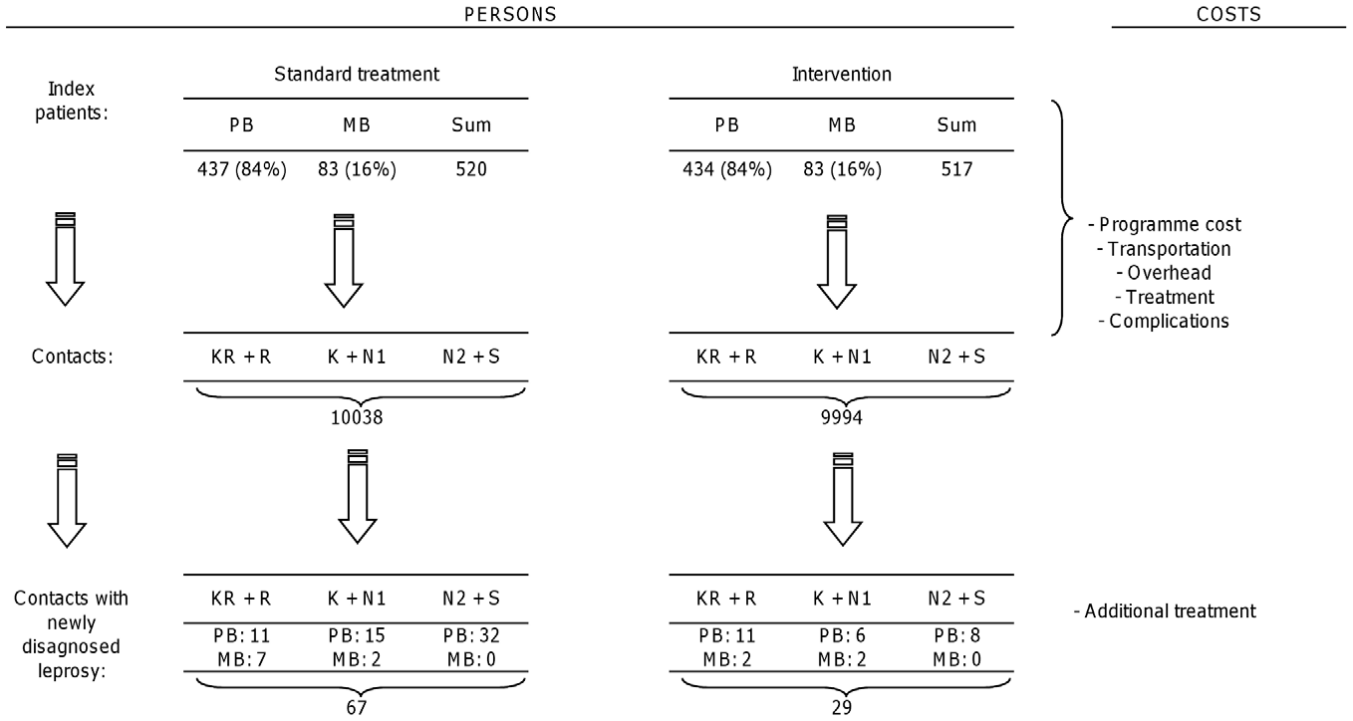
Model structure to calculate benefits (newly diagnosed leprosy patients) and costs of chemoprophylaxis with single dose rifampicin.

Cost effectiveness of the COLEP trial was assessed by calculating the incremental cost effectiveness ratio (ICER) between the standard MDT program with the additional chemoprophylaxis intervention versus the standard MDT program only. We chose the perspective of the program level, calculating the total costs of the program for the standard treatment and added per treatment arm the costs for medical treatment. The difference in treatment costs with and without the intervention was calculated and compared with the difference in the number of newly detected cases among the contacts. Costs and benefits were modelled in a decision tree [9], in which effectiveness was measured by the number of prevented leprosy cases (Figure 1). The ICER was expressed in US dollar per prevented leprosy case as follows:
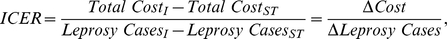
where ‘I’ denotes intervention and ‘ST’ refers to Standard treatment. Costs and effects were discounted annually with 3.5% [19].

Sub-group analyses were also carried out by distance group as defined in studies of Moet *et al*. The ICER for a specific subgroup was dependent on: i) the differences between the whole program costs, ii) the differences in the number of recipients of additional treatment and consequential costs and iii) differences in the number of new leprosy cases found in the sub-group [6], [7].

### Sensitivity analysis

To evaluate the uncertainty around the ICER, sensitivity analyses were performed by probabilistic sensitivity analysis (PSA) drawing 500 random samples. The costs and the efficiency of the program were deemed as deterministic but beta distributions were assigned to the complication probabilities to model the uncertainty around it [20]. Parameters of the beta distributions were based on the BAND study.

## Results

In total 20 032 contacts of 1037 leprosy patients remained in the trial after 2 years (taking into account loss of follow-up of 7.7%); 10 038 in the standard treatment (placebo) arm and 9 994 in the intervention (chemoprophylaxis) arm of the trial. The distributions of contacts over the two arms of the trial according to the physical distance of the contact to the index patient and the number of new leprosy cases detected in the contact groups after two years are shown in Table 2. The overall reduction of leprosy in the rifampicin arm of the trial compared to the standard treatment arm was 38 cases (57%).

**Table 2:** Number of leprosy patients arising from contacts after 2 years according to physical distance of the contacts to the index patient, by intervention (standard treatment *vs.* chemoprophylaxis).

Physical distance of contact to index	Standard treatment			Chemoprophylaxis		
	Total contacts	With leprosy		Total contacts	With leprosy	
		MB	PB		MB	PB
Household contacts (KR+R)	1 660	7	11	1 642	2	11
Next door neighbours (K+N1)	2 787	2	15	2 552	2	6
Neighbours of neighbours and social contacts (N2+S)	5 591	0	32	5 800	0	8
**Total**	**10 038**	**9**	**58**	**9 994**	**4**	**25**

Table 3 shows the total costs of different cost categories in the contact group of standard MDT treatment and of the chemoprophylaxis intervention. Program costs, which consisted of the costs of personnel, transportation and overhead, were higher for the intervention group, with around $ 4 000 due to extra personnel and transportation requirements of the program. The medical costs among the index patients of both groups amounted to approximately $ 5 700, and consisted of the treatment of leprosy with MDT and the treatment of complications. MDT treatment totalled up to $ 4 500, whereas complications estimated to have a burden of $ 1 180. The cost of complications consisted of costs of surgery and the treatment of the two known reaction types in leprosy. This was calculated as follows: unit cost of surgery was estimated to be $ 95 with a probability of need of 4.1% in MB and 1% in PB subgroups. Altogether surgery costs summed up to $ 740 in each treatment arm. The cost of reaction type 1 was $ 8, and the chance to develop such reaction was 31.7% and 2.5% in the MB and PB groups, respectively. The corresponding values for type 2 reaction were $ 71, 2% and 0%. Costs of reaction type 1 amounted to $ 300, whereas type 2 amounted to $ 142. Total cost of standard treatment was estimated to be $ 132 287, whereas chemoprophylaxis intervention needed $ 138 309 investment. The additional cost of the intervention is thus $ 6 022.

**Table 3:** Summary of total costs in the two treatment arms (in US$).

Cost category	Standard treatment	ChemoprophylaxisIntervention	Difference
1. Program	126 583	130 544	+3 961
Personnel	93 455	92 916	−539
Additional personnel	-	3 389	+3 389
Transportation	7 398	7 355	−43
Additional transportation	-	1 303	+1 303
Overhead	25 730	25 581	−149
2. Medical	5 704	5 678	−26
Treatment of leprosy	4 524	4 498	−26
Treatment of complications	1 180	1 180	0
3. Intervention (SDR)	0	2 087	+2 087
**Sum [1+2+3]**	**132 287**	**138 309**	**+6 022**

The incremental cost effectiveness ratio (ICER) indicates the cost effectiveness of the additional chemoprophylaxis intervention in contacts after 2 years versus the standard MDT treatment for all contacts together and for the three different distance groups. The ICER is expressed in US dollars saved per one prevented leprosy case. In total an incremental of $ 6 009 was invested and 38 incremental leprosy cases were prevented by chemoprophylaxis in contacts on the whole program level, resulting in an ICER of $ 158 (CI: 146–171) per one additional prevented leprosy case (Table 4). Sub-group analyses revealed that chemoprophylaxis was cost-effective for all three contact groups. It was the most cost-effective in neighbours of neighbours and social contacts (ICER $ 214), and slightly less cost-effective in next door neighbours (ICER $ 497) and least cost-effective among household contacts (ICER $ 856). Incorporation of the probabilistic aspect of the complication part into the model did not change the results considerably. The ICERs spread only in a narrow range both at the whole program level and at the sub-group level.

**Table 4:** Incremental cost effectiveness ratio's (ICER) per prevented leprosy case (in US$) of the chemoprophylaxis intervention for all contacts together and for different distance categories.

Physical distance of contact to index	Average cost-difference in US$	Difference in number of cases detected		Average ICER per case prevented(95% CI)*	
		Absolute	Discounted		
All contacts together	$ 6 009	−38	−35.5	158	(146–171)
Household contacts (KR+R)	$ 4 278	−5	−4.7	856	(762–952)
Next door neighbours (K+N1)	$ 4 472	−9	-	497	(444–553)
Neighbours of neighbours and social contacts (N2+S)	$ 5 148	−24	−22.4	214	(194–234)

*ICER at 2.5% and 97.5% of the non-parametric bootstrap sample.

## Discussion

Chemoprophylaxis with single dose rifampicin for preventing leprosy among contacts is a cost-effective prevention strategy. At program level an incremental of $ 6 009 was invested and 38 incremental leprosy cases were prevented, resulting in an ICER of $ 158 per one additional prevented leprosy case.

This is the first report on cost-effectiveness of single dose rifampicin as chemoprophylaxis in contacts of leprosy patients. The analysis is based on the results of a large randomized controlled trial in Bangladesh [6]. For the analysis, the health care perspective was taken because indirect cost data were largely unavailable. The health care perspective excludes indirect costs (patient costs), such as travel costs, loss of income due to illness and clinic visits, and long term consequences of disability. Estimating these costs was beyond the scope of this study, but inclusion would have rendered the intervention even more cost-effective. Another limitation of the study is that a static approach was taken to the analysis, measuring the effect of the intervention after two years only. After these two years, there was no further reduction of new cases in the chemoprophylaxis arm of the trial compared to the placebo arm. Because leprosy is an infectious disease, with person-to-person transmission of *M. leprae*, one can expect that prevention of primary cases (as recorded in the trial) will lead to further prevention of secondary cases. In time, this would lead to further cost-effectiveness of the intervention. Unfortunately, we could not apply such a dynamic analysis approach because there is insufficient information about the long term effects of the intervention, including the number of secondary cases prevented and the number of primary cases prevented after two years that will eventually develop leprosy after a longer period of time, beyond the 4 years observation period of the trial.

It is also important to understand that the results of the COLEP trial reflect a comparison between the chemoprophylaxis intervention and standard MDT treatment plus contact surveys at 2-year intervals with treatment of newly diagnosed cases among contacts. A contact survey in itself is an intervention that reduces transmission in contact groups and thus new leprosy patients among contacts. The provision of chemoprophylaxis to contacts requires contact tracing, but contact tracing is not part of leprosy control programs in many countries and doing so would increase program costs considerably. WHO however, recognizes the importance of contact tracing and now recommends that it is introduced in all control programs [21]. This would then also lay a good foundation for introducing chemoprophylaxis.

WHO reports regarding cost-effectiveness analyses recommend using disability adjusted life years (DALY) as outcome measure for such studies [22]. In leprosy two measures are common to express disability: WHO grade 1 and 2 [23]. The disability weight for grade 2 disability (visible deformity) has been determined at 0.153 [24], but no weight is available for grade 1. Of all newly detected leprosy cases, a relatively low percentage (2–35%) have grade 2 disability [25]. In our study we chose for the number of leprosy cases prevented as outcome, because there is little information available about survival of patients with grade 2 disability and also because the choice for DALY's would have given a less favourable result due to the low weight of leprosy disability.

There are a number of issues to take into account when relating the outcome of this study to other countries. Firstly, the cost level to conduct leprosy control will differ per country, due to economic standard, budget allocated to primary health care, salaries of health care workers, etc. In our calculation, program costs were similar for both the standard MDT treatment and chemoprophylaxis intervention, but these costs will vary per country. The treatment costs are based on real cost estimates and will vary less between countries and programs. Therefore the actual costs will differ, but the conclusion that the intervention is cost-effective is very likely to remain the same. Secondly, the clinical presentation of leprosy differs between countries and regions. Globally the distribution is around 40% for MB and 60% for PB in newly detected leprosy cases, but with widely varying ratios between countries [25]. Since costs for treating PB and MB leprosy are different, these differences are likely to affect the outcome of the cost-effectiveness analysis. Thirdly, the percentage of newly detected cases that are a household contact of a known leprosy patient differs per country and is possibly determined by the endemicity level of leprosy in a country or area. In Bangladesh, in the high endemic area where the COLEP study was conducted, approximately 25% of newly detected cases had a known index case within the family, whereas in a low endemic area (Thailand) this proportion was 62% [26]. An intervention aimed at close (household) contacts may therefore be more cost-effective in countries where relatively many new cases are household contacts. But the background and implications of such differences on effectiveness of chemoprophylaxis needs further research.

Only few articles have been published about cost-effectiveness analyses of interventions in leprosy [27]. Most articles assess small parts of leprosy control, such as footwear provision [28], MDT delivery costs [29], or the economic aspects of hospitalisation versus ambulatory care of neuritis in leprosy reactions [30]. Only two studies provided a more general cost-effect analysis. Naik and Ganapati included several costs in their economic evaluation, but a limitation of the study is the lack of reference about how they obtained their cost data [31]. Remme *et al.* based the cost calculations in their study on the limited available published cost data, program expenditure data and expert opinion, and also provide limited insight into how they obtained certain costs and effects [30]. Both studies do not mention well how the costs are obtained, (e.g. real costs, bottom-up or top-down costs). Our current article is basically one of the first structured cost-effective analyses for leprosy presenting an overview of the costs involved and can be used for the assessment of the costs of leprosy control in general.

This report shows that chemoprophylaxis with single dose rifampicin given to contacts of newly diagnosed leprosy patients is a cost-effective intervention strategy. Implementation studies in the field are necessary to establish whether this intervention is acceptable and feasible in other leprosy endemic areas of the world.
